# Chemotherapy Extravasation Causing Soft-Tissue Necrosis Mimicking Infection: A Longitudinal Case Study

**DOI:** 10.7759/cureus.55333

**Published:** 2024-03-01

**Authors:** Michael Nguyen, Luke Borders, Jacqueline T Wesolow, John Greene

**Affiliations:** 1 Internal Medicine, Morsani College of Medicine, University of South Florida Health, Tampa, USA; 2 Internal Medicine, Moffitt Cancer Center, Tampa, USA

**Keywords:** extravasation injury, soft tissue necrosis, chemotherapy extravasation, necrotizing, cancer patient

## Abstract

Extravasation injuries are uncommon, underreported, and often misdiagnosed in patients. The signs and symptoms of extravasation injuries vary from simple pain and tenderness to tissue necrosis and potentially fatal secondary infections. Extravasation may progress to more severe conditions such as necrotizing fasciitis (NF) or cellulitis, so special care is needed by physicians to identify and treat these injuries correctly. Here, we explore a case study on extravasation injuries mimicking NF leading to infectious complications and discuss the proper diagnosis and treatment of extravasation injuries as well as other NF-mimicking diseases. We present a case of a 44-year-old Hispanic male with a history of B-cell acute lymphoblastic leukemia who underwent inpatient chemotherapy treatment via a chest port.

## Introduction

Extravasation is defined as the leakage of a solution being delivered intravenously into neighboring tissue. The range and magnitude of symptoms due to extravasation depend on the class of medication effluxed but can range from erythema, edema, and pain to tissue necrosis [1]. The sequelae of extravasation injuries can delay treatment and can be a potential route for infection. Chemotherapeutics are particularly noteworthy in extravasation injuries due to their ability to damage cell DNA and long half-life in tissue [2,3]. Extravasation of chemotherapeutics is uncommon, with a published incidence of 0.1%-6%, but may be higher due to underreporting [1]. We present a case of soft-tissue injury secondary to chemotherapy extravasation involving an immunocompromised cancer patient that mimicked necrotizing cellulitis.

## Case presentation

**Figure 1:**
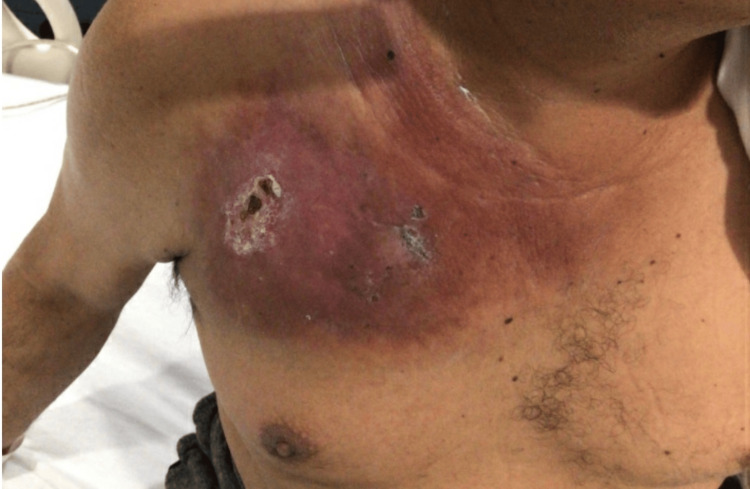
Initial area of extravasation causing a semicircular erythematous lesion around the area of the chest port.

**Figure 2:**
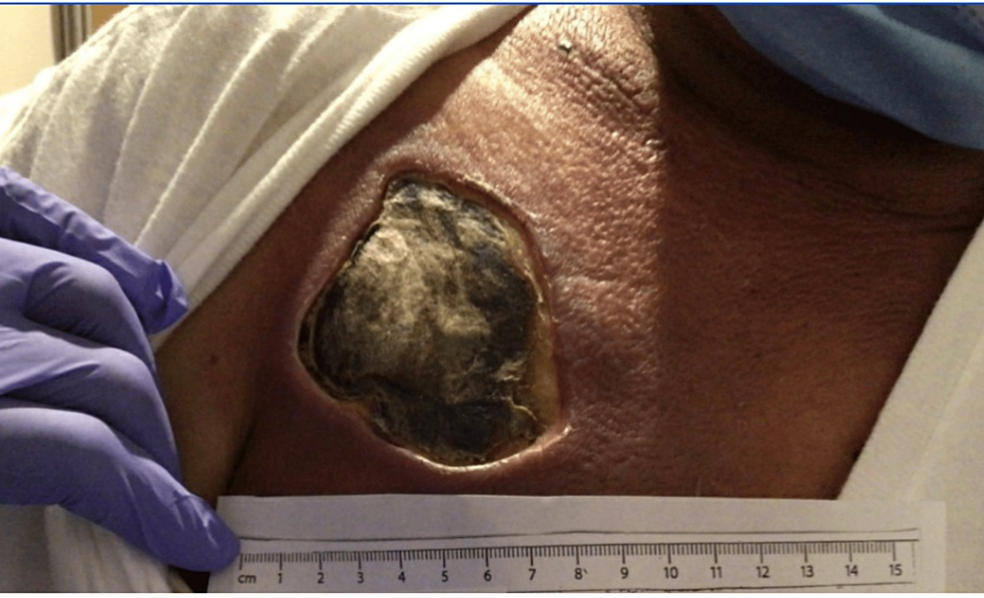
Removal of chest port and overlying skin.

 A 44-year-old Hispanic male with a history of B-cell acute lymphoblastic leukemia underwent inpatient chemotherapy treatment via a port in his right chest. While hospitalized at an external facility, it was observed that the tissue surrounding his chest port became painful and discolored, eventually progressing to necrosis over several days due to chemotherapy extravasation (Figure 1). The initial area of extravasation was noted to be a semicircular erythematous lesion affecting the top layer of skin measuring about 14 cm in diameter. Subsequently, the wound developed cellulitis and myositis, necessitating debridement and eschar removal (Figure 2). Wound closure was postponed to accommodate ongoing leukemia chemotherapy. Cultures obtained from the wound revealed the presence of a nonsporulating dematiaceous mold, which was successfully treated with oral voriconazole for one month. Extravasation injuries are rare intravenous (IV) site complications, making this a unique case.

**Figure 3:**
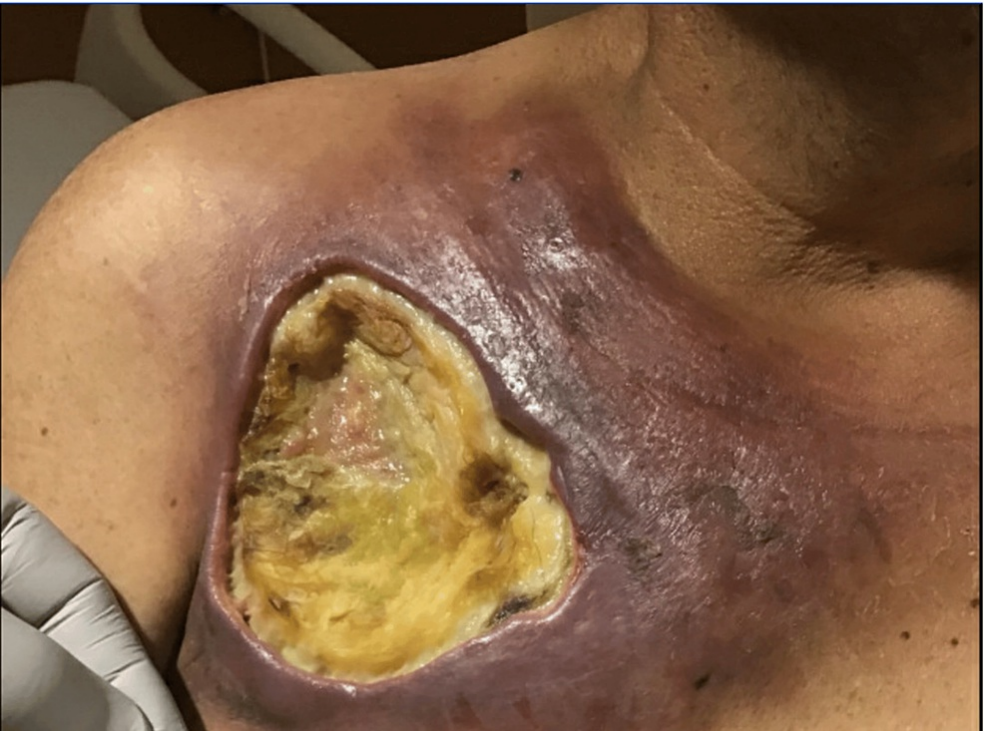
Further surgical debridement of the surrounding skin, fat, and fascia.

Months later, the open chest wound in the setting of neutropenia developed cellulitis and myositis. Wound debridement and cultures of the chest tissue grew two strains of *Escherichia coli* resistant to ceftriaxone, trimethoprim/sulfamethoxazole, and ciprofloxacin, and one strain resistant to cefepime and intermediate to minocycline. Later, a third debridement was required, and the cultures taken grew *E. coli* resistant to cefepime, cefoxitin, Cipro, trimethoprim/sulfamethoxazole, intermediate to piperacillin/tazobactam, and sensitive to meropenem and ertapenem. Further wound debridement with no antibiotic treatment for the isolated bacteria was performed during this period. The patient was not neutropenic (Figure 3).

**Figure 4:**
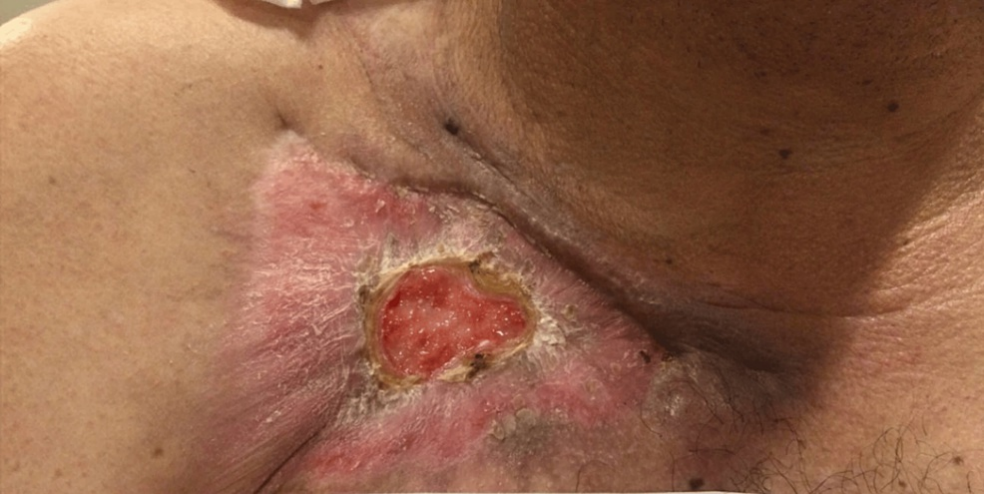
Chest wound approximately one year following initial extravasation injury.

One month later, he was admitted with a necrotic right neck furuncle. He denied cough, congestion, abdominal pain, nausea, vomiting, diarrhea, dysuria, or new rashes (aside from the neck lesion). The patient reported no change in pain in the wound on his right chest. Vitals taken at the time showed a temperature of 98 °F, blood pressure of 128/86 mmHg, pulse rate of 69 beats per minute, respirations of 18 breaths per minute, weight of 83.7 kg, 31.1 body mass index (BMI), and 100% saturation on room air. Labs are shown in Table 1. HIV, hepatitis B, hepatitis C, and Strongyloides antibodies were all negative. The neck wound was incised, drained, and grew methicillin-resistant *Staphylococcus aureus* (MRSA), and it was treated with linezolid for one week. Following therapy, the patient was sent home with a wound vac awaiting flap closure of the chest wound. Over the next three months, the wound healed with a covering of new skin and did not need surgical closure (Figure 4). In addition, the patient experienced a relapse of leukemia, necessitating further cycles of chemotherapy. This was complicated by neutropenic fever and nodular pneumonia, presumed to be mold pneumonia. The small chest wound eschar had minimal drainage that was cultured and grew *Enterobacter cloacae* resistant to Cipro and trimethoprim/sulfamethoxazole. He received vancomycin, cefepime, liposomal amphotericin B, and acyclovir. Bronchoscopic alveolar lavage remained negative for one day.

**Table 1:** Laboratory data.

Lab	Patient’s value	Normal range
White blood cell (WBC)	5.9 k/uL	4.5-11 k/uL
Hemoglobin	11.1 g/dL	13.2-16.6 g/dL
Platelets	126 k/uL	150-450 k/uL
Lymphs	1.14 k/uL	1.1-3.5 k/uL
Blood urea nitrogen (BUN)	11 mg/dL	6-23 mg/dL
Creatinine	0.4 mg/dL	0.5-1.0 mg/dL
Glucose	130 mg/dL	70-110 mg/dL

## Discussion

Extravasation is an uncommon phenomenon in the developed world. It has been an extensively reported topic, and we have guidelines to prevent and manage that. Our patient presented with a rare extravasation injury secondary to his IV chemotherapy. Extravasation injuries present similarly to necrotizing cellulitis and, therefore, can be missed; therefore, it is imperative to identify them early on. Common sites for extravasation injury include regions with diminished amounts of soft tissue and regions with insufficient venous flow. Insufficient venous flow increases the probability of pooling and exudating agents near the cannula [1]. Extravasation of IV fluids initially presents with pain and swelling but then may evolve into blistering or discoloration. Additional symptoms may include induration that usually progresses into a dark black eschar, which can subsequently flake off to unveil underlying subcutaneous tissue ulceration.

Extravasation complications have a broad spectrum of presentations. For example, Cisplatin, an anticancer agent, leads to tissue necrosis when administered in higher concentrations and usually elicits inflammatory responses such as swelling and inflammation at lower concentrations [4]. Other factors that potentially increase extravasation complications include the drug's binding ability to DNA, the drug’s lethality to replicating cells, the drug’s propensity to dilate vessels, the pH of the drug, and the drug’s formulation parameters [1].

In the context of extravasation, drugs can either be vesicants or irritants. Leakage of irritants is typically less severe, producing localized symptoms such as pain, tightness, inflammation, and phlebitis at the cannulation site or associated vein. These symptoms are usually self-limiting, rarely cause long-term complications, and do not result in tissue necrosis. In contrast, extravasation of vesicants is a much more dangerous event, as vesicants may cause irreversible blistering, sloughing of tissue, and tissue necrosis when leaked [5].

Extravasation of irritants is known to mimic cellulitis through a variety of mechanisms. Hyperosmolar agents will shift water out of cells, which can lead to cellular transport dysregulation, reactive oxygen species (ROS) production, and ultimately, cell DNA damage and apoptosis [5,6]. This leads to symptoms such as dermal thickening, swelling, erythema, and pain, all of which are common in cellulitis. While generally localized to the skin, cellulitis can progress to necrotizing fasciitis (NF), sepsis, osteomyelitis, or other complications, depending on the location of the infection [7]. Both cellulitis and NF present with soft-tissue swelling on CT and MRI [8]. 

Extravasation risk can also be lumped into two significant factors: infusion-based and patient-based. Infusion-based factors are induced by aberrant operation of the cannula or by infusion characteristics. Re-accessing veins from previous attempts, constant catheter adjustment, and increased flow rate are all risk factors for extravasation [6,9]. Patient-based risk revolves around the characteristics of the patients. The patient's skin tone may affect the speed of injury detection. Hypotension has proven to raise chances of extravasation as well. Poor venous blood flow may cause local vessel pooling, thus resulting in spasm of the vessel and leakage of fluid into encompassing tissues [6,9,10]. This spasm mechanism is prominent in individuals with Raynaud disease [6]. It should be noted that a patient with prior extravasation injury is more likely to experience another bout of extravasation if additional cannulation is required. A patient’s tissue strength also plays a role in patient-based risk. Clot development near the area of access is another sign to note, as most extravasations are stimulated by backflow. Patients with lymphedema or who are elderly or neonates pose a high risk of extravasation [6,9,10]. Patients with altered mental status are also at a greater risk as they may pull on or tug cannulas. Similarly, patients with sensory or communication deficits cannot point out symptoms such as pain that may suggest a diagnosis of extravasation [6].

Treatment of extravasation injury primarily depends on the progression of the injury. Injury progression is heavily influenced by IV solution type [1]. Regardless of the solution type, the infusion should be halted immediately. An acute episode warrants prompt emergency medical attention and aspiration of the fluid from the cannula. Pooling fluid in tissues should also be liberated. If a limb is used as the site of cannulation, the affected limb should be immobilized and raised above heart level to aid in lymphatic drainage [1,3,11]. Conservative treatment is generally preferred. However, expedited debridement following wound manifestation may be preferred in cases where treatment of the principal diagnosis cannot be delayed [11-13]. Vesicant extravasation is treated in alignment with the methods mentioned above. Cold packs may also be used to combat pain, and hot packs increase vasodilation and are also effective at minimizing solution concentration [1,3]. Prevention of extravasation relies largely on taking proper precautions and attentively monitoring patients’ overall health. It begins with the mindful placement of all venous cannulas, followed by a brief, sterile saline rinse [1,14]. Dressing is highly encouraged to prevent movement of the cannula. The cannulated area should be checked on an hourly basis, and the dressing should be minimal so as not to hide skin susceptible to signs of extravasation [1,14]. Concentrated solutions and solutions with highly deviated pHs should be administered through a central line. This also applies to solutions with vesicant or irritant tendencies, and in addition, these solutions can also be diluted. The preferred area for cannula insertion is typically on the forearm or dorsal portion of the hand [1,14]. This preference is because of the availability of access and ease of monitoring. Cannulation should be avoided near joints as an increased likelihood of damage to tendons or nerves may occur. A peripherally inserted central catheter line or central line is best suited for the slow administration of drugs with more significant potential to cause extravasation [1,14]. If a variety of solutions are required, the drug with the most remarkable propensity to cause extravasation should be encouraged first. If any doubts or confusion arise, discontinuation of the infusion and a change in the cannula site may be necessary if cannulation must resume [1,14].

Many drugs are known to cause extravasation injuries, with the primary mechanism of action of the drug being the chief risk factor. Medications with increased extravasation rates include anticoagulants, antifibrinolytics, antiplatelets, vasodilators, hormone therapy drugs (including steroids), diuretics, antihistamines, analgesics, and IV antibiotics [1]. The first three medications produce similar responses; anticoagulants, antifibrinolytics, and antiplatelets may influence extravasation by increasing localized bleeding. Vasodilators, along with hormone therapy drugs, steroids, and diuretics, all possess vasodilating capabilities and may increase the area of injury [1]. Antihistamines function to constrict vessels and enhance the probability of ischemic complications. Repeated IV antibiotic injection can irritate the vessel, thus providing a higher chance of thrombosis. Although analgesics do not directly cause extravasation, they dampen the pain of nascent extravasation symptoms, resulting in a decline in symptom awareness [1]. 

Differential diagnosis 

Because extravasation injuries are rare IV site complications, as in our case, the differential diagnosis involves ruling out other complications that present with necrosis at the insertion site. Sweet’s syndrome, NF, and pathergy are all conditions that can induce necrosis at the sight of canulization and, therefore, must be ruled out when considering a diagnosis of extravasation injury [15].

Sweet’s syndrome is an uncommon inflammatory complication that arises through various etiologies, with symptoms including erythema-associated papules, plaques, or nodules [16]. Criteria for a complete diagnosis of Sweet’s syndrome include fever greater than 38 °C, possession of a malignant tumor, pregnancy, infection, or inflammatory disease, and abnormal bloodwork such as neutrophilia (>70%). Sweet’s syndrome shows dense neutrophilic infiltration of the dermis upon histopathologic analysis. Discerning between the diagnosis of Sweet’s syndrome and other similar diagnoses relies primarily on the expectation of suspected lesions. Effective treatment and disappearance of lesions with corticosteroids further point toward the diagnosis of Sweet’s syndrome. Sweet’s syndrome is also noted to present at sites of IV chemotherapy and is associated with pathergy, hence making its presentation similar to drug extravasation [17,18]. Sweet’s syndrome can also mimic necrotizing fasciitis, presenting clinically and histopathologically in a similar manner. Necrotizing Sweet’s syndrome differs from classical Sweet’s syndrome in that neutrophilic infiltration goes past the epidermis and subcutaneous fat and into the fascia and skeletal muscle [15].

NF is a feared complication for any IV site or wound, including those caused by extravasation, due to its high mortality rate. However, only seven cases of chemotherapy extravasation causing NF have been reported [5,6]; therefore, it is more common for chemotherapy extravasation injuries to present with sequelae that mimic NF. The key diagnostic finding for NF is the presence of gas on imaging. CT scanning is the preferred modality because it is faster, not overly sensitive, and thus better at identifying gas when compared to MRI [7,8]. Common causes of NF include Streptococcal infections, occasionally *S. aureus*, and the progression of cellulitis [16]. Treatment of NF involves rapid identification of the involved pathogens, susceptibility tests, and antibiotic management [2,4]. Surgical intervention may be required depending on the degree of infection, with improved survivability if surgical intervention is initiated within 24 hours of hospital admission [19].

Skin pathergy reaction (SPR) is a hyperreactive inflammatory response following needle-induced skin trauma. Pathergy is characterized by the formation of pustules and papules 24 to 48 hours following skin trauma. The presence of pathergy is vital for the diagnosis of Behçet's disease. While the exact methodology for diagnosing SPR is still debated, the general consensus is that a positive SPR involves the development of an erythematous papule or pustule >2 mm in diameter 24-48 hours following the skin prick [17].

SPR is often associated with Pyoderma granulosum (PG), an aseptic skin disease that can also produce necrotic ulcers. Similar to SKR, in 25% to 50% of PG cases, it has been shown that trauma or surgery is the triggering factor. However, the majority of PG cases are associated with a systemic disease such as inflammatory bowel disease, arthritis, or another hematologic disorder. The key distinction of PG from other necrotic ulcer-inducing pathologies is one or more burrowing ulcers with an irregular margin and a ragged purple-red overhanging edge. Histologic samples of the ulcer are necessary to rule out PG [18].

Primary cutaneous mold infections can also develop at central and peripheral IV catheter insertion sites or colonize and infect a necrotic wound. While most cutaneous mold infections result from traumatic inoculation contaminated with soil, other sources in a clinical setting include cannulation sites, elasticized bandages, wound dressings, and medical tape [19]. The most common mold infections are zygomycosis, aspergillosis, and phaeohyphomycosis.

Phaeohyphomycosis is an infection caused by dematiaceous (melanized) fungi. Melanin acts as a virulence factor as it prevents phagocytosis of the fungi. Infections are typically present as subcutaneous cysts but can range from superficial lesions to systemic infections [20,21]. Most infections result from traumatic inoculation with contaminated wood or soil, but immunocompromised patients are particularly at risk of developing systemic infections [20]. All-cause mortality of patients with only superficial or subcutaneous lesions is around 30% but is nearly doubled in patients with systemic infections [21]. The most common fungi responsible for phaeohyphomycosis are *Exophiala jeanselmei*, *Scedosporium prolificans*, *Exophiala* spp., and *Alternaria* spp. [22].

Diagnosis of phaeohyphomycosis traditionally involves histopathologic analysis and drawing cultures to isolate the exact species of fungi. However, recent studies have shown that polymerase chain reaction (PCR) assays may be a superior modality as they are faster and more sensitive and can quantify the fungal burden of patients [20]. Treatment of phaeohyphomycosis involves surgical debridement of the wound and the use of azole antifungal medications such as itraconazole, voriconazole, posaconazole, caspofungin, and amphotericin B. In our case, our patient developed a large eschar for which prompt debridement and one month of voriconazole were enough to cure the infection with no recurrence [20]. Systemic infections are similarly treated with the aforementioned antifungals, but studies have shown that tailoring the antifungals to specific species of fungi can improve outcomes [23].

Our case is similar to another reported case in the literature where a 47-year-old female developed a rash following IV medication administration [24]. Two days later, a blister formed, and after three weeks, the wound sloughed. The patient underwent wound debridement with vacuum dressing [24]. This vacuum dressing and debridements were continued until the wound was able to be closed [24 ]. This case and our case stress the importance of early recognition of extravasation injuries to prevent further skin and underlying tissue damage.

## Conclusions

Extravasation of chemotherapy in leukemia patients results in the infiltration of the chemotherapy solution from a vessel into neighboring tissues. This can cause severe tissue damage, leading to cellulitis, which may progress to myositis. Treatment typically involves wound debridement. Ultimately, prevention of extravasation complications relies strongly on proper precaution and attentive monitoring of the patient’s status. Many drug classes increase susceptibility to extravasation, and knowledge of these possibilities allows for the reduction of complications. Sweet's syndrome, NF, SKR, and pathergy, all present similarly to extravasation injury as they all can induce necrosis at IV sites. Therefore, when a patient presents with necrosis secondary to a suspected extravasation injury, histopathological analysis of the site of injury, imaging, cultures, and a skin prick test are all necessary to rule out Sweet's syndrome, NF, SKR, and pathergy, respectively.
